# The global scientific evolution of cardiopulmonary performance and threshold research in sports sciences during 2010–2025: a comparative bibliometric analysis based on web of science and scopus

**DOI:** 10.3389/frma.2026.1905302

**Published:** 2026-08-03

**Authors:** Mehmet Soyal, Ömer Aksoy, Nuri Muhammet Çelik, Meltem Özaǧır, Ünal Can Gökmen, Uǧur Caba

**Affiliations:** 1 Department of Coaching Education, Faculty of Sports Sciences, Istanbul Gelisim University, Istanbul, Türkiye; 2 Department of Coaching Education, Faculty of Sports Sciences, Batman University, Batman, Türkiye; 3 Department of Recreation, Faculty of Sports Sciences, Istanbul Gelisim University, Istanbul, Türkiye; 4 Department of Exercise and Sport Sciences, Faculty of Sports Sciences, Istanbul Gelisim University, Istanbul, Türkiye

**Keywords:** cardiopulmonary performance, lactate threshold (LT), sports science, ventilatory threshold (VT), VO_2_max

## Abstract

**Objective:**

Cardiopulmonary performance and physiological threshold parameters, including VO_2_max, lactate threshold, ventilatory threshold, and respiratory compensation point, are central determinants of endurance capacity and athletic performance. Despite the growing volume of research in this area, the global scientific structure, conceptual evolution, and collaborative dynamics of this literature have not been systematically synthesized. This study aimed to reveal the global scientific evolution, publication dynamics, structural concentration, and conceptual patterns of cardiopulmonary performance and threshold research in sports sciences between 2010 and 2025 through a comparative bibliometric analysis based on Web of Science and Scopus.

**Methods:**

A bibliometric analysis was conducted using records retrieved from the Web of Science Core Collection and Scopus databases. After database filtering, merging, and strict duplicate removal based on normalized DOI and title matching, a final dataset of 2,745 unique original articles was analyzed. The analytical framework included descriptive bibliometric indicators, annual scientific production trends, compound annual growth rate (CAGR), regression-based trend analysis, Bradford's Law, Lotka's Law, keyword co-occurrence network analysis, thematic mapping, country collaboration network analysis, and citation half-life assessment.

**Results:**

The findings demonstrated a sustained growth trajectory in the literature, with annual publication output increasing from 123 articles in 2010 to 276 in 2025 and a CAGR of approximately 5.53%. Regression analysis confirmed a strong and systematic upward trend (*R*^2^ = 0.81). Bradford distribution revealed a distinct core–periphery journal structure, with only seven journals accounting for 31.3% of total publications. Keyword co-occurrence and thematic map analyses showed that the field is conceptually structured around physiological determinants, particularly exercise, oxygen consumption, VO_2_max, anaerobic threshold, and ventilatory threshold. VO_2_max and exercise performance emerged as motor themes, whereas ventilatory threshold and lactate threshold appeared as basic themes, indicating their foundational but still evolving role in the field. Country collaboration analysis demonstrated a dense and internationally connected research network, led primarily by the USA and several European countries. Citation half-life findings indicated that the field maintains both current relevance and cumulative continuity, with a median cited reference year of 2018.

**Conclusion:**

The results indicate that cardiopulmonary performance and threshold research in sports sciences has evolved into a structurally consolidated, conceptually centralized, and internationally collaborative scientific field. The literature is strongly organized around physiological threshold-based mechanisms rather than isolated performance outcomes, reflecting a mature yet still expanding research architecture. By integrating production trends, conceptual mapping, and collaboration patterns, this study provides a comprehensive overview of the information structure of the field and offers a robust framework for future research in sports physiology and performance science.

## 1 Introduction

Cardiopulmonary performance is defined as the capacity to take up oxygen, deliver it to different tissues, and utilize it during exercise and is considered a key parameter for determining many aspects of athletic performance. In particular, maximum oxygen consumption (VO_2_max) is recognized as an international “gold standard” for assessing cardiopulmonary function (Hill and Lupton, 1923). It is not merely a quantitative indicator of maximal oxygen consumption; rather, it is considered as a functional capacity resulting from the interaction of several physiological components, such as cardiac output, stroke volume, hemoglobin levels, capillary density, and intramuscular oxidative capacity (Bassett, 2000). Measuring this capacity plays an essential role in processes, such as assessing athletes' motor skills and designing workout programmes (Ross et al., 2016; Armstrong and Welsman, 2019). Differences in performance among individuals stem not only from maximal oxygen uptake, but also from how much of that capacity can be used and how long it can be sustained (Coyle, 1995). Research on the determinants of endurance performance has revealed that, besides VO_2_max, the lactate threshold and exercise economy must be assessed together (Joyner and Coyle, 2008). Therefore, threshold workouts aim to enhance the body's oxygen efficiency in order to utilize this capacity to a greater extent and for a longer duration (Laursen and Jenkins, 2002). In this sense, the lactate threshold (LT) is defined as the point at which blood lactate concentration begins to rise significantly above the resting level as exercise intensity increases. LT refers to the point at which intramuscular metabolic stress increases and anaerobic contribution grows relatively (Faude et al., 2009). The ventilatory threshold (VT) represents the stage where ventilation begins to rise disproportionately relative to oxygen consumption as well as a breakpoint in respiratory parameters. VT indicates the point at which respiratory compensation begins against metabolic acidosis associated with activity-induced lactate accumulation. It is therefore physiologically linked to LT but is measured using a different method; LT is measured via blood analysis, whilst VT is measured via gas exchange analysis (Wasserman et al., 2012). The respiratory compensation point (RCP), on the other hand, is described as the second inflection point following the ventilatory threshold and the final limit of the sustainability of vigorous-intensity exercise. At this stage, the rise in ventilation becomes even more pronounced, and carbon dioxide excretion accelerates in order to compensate for metabolic acidosis (Beaver et al., 1986). Workloads at the threshold zone increase mitochondrial density, enhance oxidative enzyme activity, and promote intramuscular metabolic adaptations (Burgomaster et al., 2006; Howarth et al., 2009). Furthermore, the literature reports that even though VO_2_max values are similar among elite athletes, threshold values are a significant factor that distinguishes performance (Faude et al., 2009). Recent literature has increasingly emphasized that VO_2_max should not be interpreted as an isolated maximal indicator, but together with sustainable metabolic capacity, threshold-derived intensity domains, and sport-specific physiological responses. In contemporary endurance training, intensity zones are commonly anchored below, between, and above the first and second metabolic or ventilatory thresholds. However, the terminology used to describe these boundaries remains inconsistent across studies. Terms such as aerobic threshold, anaerobic threshold, LT1, LT2, VT1, VT2, and respiratory compensation point are sometimes used interchangeably or with different physiological meanings, creating conceptual ambiguity in both research and applied training prescription (MacIntosh et al., 2021; Mazaheri et al., 2021; Casado et al., 2022).

It has been reported that athletes with similar VO_2_max values may perform differently in competitions and a significant portion of this difference is related to their threshold values (Bassett, 2000). Therefore, threshold studies have occupied a significant place in training planning and research in recent years (Midgley et al., 2007). Studies in the literature have reported that threshold training significantly improves cardiopulmonary performance by increasing the maximal oxygen uptake of the organism (Helgerud et al., 2007). In particular, the role of maximal oxygen consumption and threshold parameters in explaining performance capacity has been emphasized in many studies in the literature (Jones and Vanhatalo, 2017; Poole et al., 2016).

Given the rapid expansion of threshold-based performance research, the coexistence of multiple threshold terminologies, and the emergence of automated and wearable assessment approaches, bibliometric mapping is needed to clarify how this literature is conceptually organized, where it is fragmented, and which methodological debates have shaped its evolution. Bibliometric studies examine the quantitative aspects of scientific knowledge and allow researchers to evaluate publication patterns, citation structures, and thematic development within a field (Ellegaard and Wallin, 2015; Donthu et al., 2021). This type of analysis is regarded as a suitable method for evaluating research results based on statistical tools and for examining the measurable characteristics of knowledge produced in a specific field (Ellegaard and Wallin, 2015). Bibliographic literature has gained significance in recent years due to digitisation, the systematization of information, and the generation of numerous and diverse scientific literature databases accessible electronically worldwide (Ayala et al., 2010). Thus, it provides researchers with a comprehensive understanding of the field's historical development and current state, whilst offering insights into current topics and research trends (Bornmann and Leydesdorff, 2014; Zhang et al., 2022). The literature includes studies prepared using bibliometric analysis method on various topics such as the effects of exercise on health (Zhang et al., 2022), exercise and the cardiovascular system (Lavie et al., 2015), and the improvement of sporting performance.

This study aimed to reveal the global evolution, publication dynamics, structural concentration, and conceptual patterns of research on cardiopulmonary performance and physiological thresholds in the field of sports science during 2010–2025, from a bibliometric perspective. As such, the study is expected to bridge an important gap in the existing literature by highlighting publication trends and keyword dynamics within the field and to offer original contributions that will serve as a guide for future research.

During 2010–2025, research on cardiopulmonary performance and threshold in sports science has generally focused on physiological outcomes. This study aims to bridge the gap in the literature by examining the conceptual and structural evolution of the concepts of cardiopulmonary performance and threshold in a comparative manner, using both WoS and Scopus. In the study, it is hypothesized that the number of annual publications on cardiopulmonary performance and physiological threshold studies has increased significantly over the last 15 years. A bibliometric analysis provides a suitable framework for understanding the distribution of scientific output in studies on cardiopulmonary performance and threshold measurements in sports sciences during 2010–2025, as well as the conceptual trends that have developed in the field. Whilst numerous studies in the field of sports sciences focus on specific performance parameters or physiological models, this study systematically lays out the structural organization and knowledge architecture of the research topic.

## 2 Method

### 2.1 Research design

This study is designed as a bibliometric analysis based on a quantitative research approach to examine holistically the scientific development process of research on cardiopulmonary performance and threshold within the field of sports sciences. The bibliometric method allows researchers to identify the publication output trends, citation dynamics, knowledge dissemination networks and conceptual structures within a specific scientific field through objective indicators. Accordingly, the study is founded on a multi-layered model that integrates descriptive, structural, and network-based analyses.

### 2.2 Data sources and search strategy

**Figure 1:**
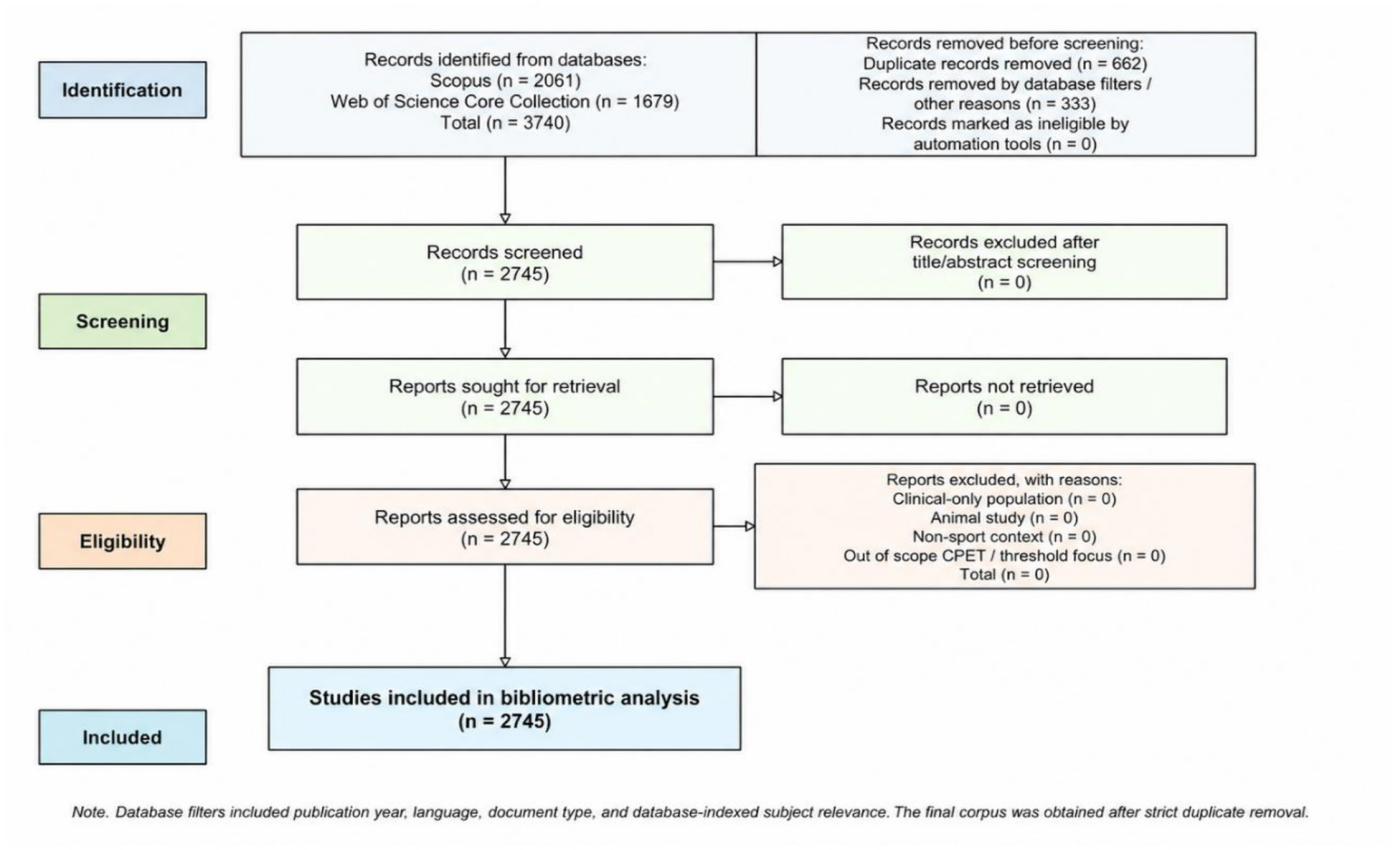
PRISMA 2020 flow diagram for bibliometric corpus construction.

The literature search was conducted in the Scopus and Web of Science Core Collection databases. The combined use of these two databases was preferred to reduce database coverage bias and to provide a broader representation of the literature. The Scopus search was performed in the TITLE-ABS-KEY field, whereas the Web of Science search was performed in the Topic (TS) field. The search was restricted to publications published between 2010 and 2025, written in English, and indexed as article-type records. The initial search yielded 2,061 records in Scopus and 1,679 records in Web of Science Core Collection. After applying database filters for publication year, language, document type, and database-indexed subject relevance, 1,849 Scopus records and 1,558 Web of Science records remained. The filtered records were exported and merged in the R environment. Duplicate records were removed using normalized DOI matching and normalized title matching. After strict duplicate removal, 662 duplicate records were removed and 2,745 unique records were retained for bibliometric analysis. Because this study was designed as a bibliometric corpus construction rather than a full systematic review of intervention effects, records remaining after database filtering and duplicate removal were retained for bibliometric analysis. The selection process was reported using a PRISMA 2020 flow diagram adapted for bibliometric corpus construction (Page et al., 2021). The bibliometric corpus construction and record-selection process are presented in Figure 1.

Scopus: TITLE-ABS-KEY ((“cardiopulmonary exercise test^*^” OR CPET OR “cardiorespiratory fitness test^*^” OR “exercise test^*^” OR “incremental test^*^” OR “ramp test^*^” OR “ramp protocol^*^”)AND(athlete^*^ OR sport^*^ OR “trained individual^*^” OR “elite athlete^*^” OR “endurance athlete^*^” OR runner^*^ OR cyclist^*^)AND(“anaerobic threshold” OR “aerobic threshold” OR “lactate threshold” OR “ventilatory threshold” OR “respiratory compensation point” OR RCP OR “isocapnic buffering” OR VO2max OR “VO2 max” OR “VO_2_max” OR VO2peak OR “VO2 peak” OR “maximal oxygen uptake” OR “peak oxygen uptake”)) AND PUBYEAR > 2009 AND PUBYEAR < 2026AND (LIMIT-TO(DOCTYPE, “ar”)) AND (LIMIT-TO(LANGUAGE, “English”)).

WOS: TS = ((“cardiopulmonary exercise test^*^” OR CPET OR “cardiorespiratory fitness test^*^” OR “exercise test^*^” OR “incremental test^*^” OR “ramp test^*^” OR “ramp protocol^*^”)AND(athlete^*^ OR sport^*^ OR “trained individual^*^” OR “elite athlete^*^” OR “endurance athlete^*^” OR runner^*^ OR cyclist^*^)AND(“anaerobic threshold” OR “aerobic threshold” OR “lactate threshold” OR “ventilatory threshold” OR “respiratory compensation point” OR RCP OR “isocapnic buffering” OR VO2max OR “VO2 max” OR “VO_2_max” OR VO2peak OR “VO2 peak” OR “maximal oxygen uptake” OR “peak oxygen uptake”)).

Refined by:

Publication years = 2010–2025

Document type = Article

Language = English

The exact Boolean search syntax used in Scopus and Web of Science is provided in Supplementary Table 1.

### 2.3 Inclusion criteria

- Published between 2010 and 2025,
- Published only in English,
- Being a peer-reviewed research paper in the “Article” type,
- Relevance to sports science, athletic performance, exercise testing, cardiopulmonary performance, VO_2_max, or physiological threshold parameters as identified through the search strategy and database-indexed fields,
- Inclusion of cardiopulmonary exercise testing and physiological threshold parameters (VO_2_max, anaerobic threshold, ventilatory threshold, RCP, isocapnic buffering).

### 2.4 Exclusion criteria

Exclusion criteria were:

- duplicate records;
- records outside the 2010–2025 publication period;
- non-English records;
- non-article document types, where identified by database filters;
- records without sufficient bibliographic metadata for analysis.

Since this study was a bibliometric corpus construction, eligibility was primarily operationalized through database filters, search-field relevance, and duplicate removal rather than full-text methodological appraisal.

### 2.5 Data collection and extraction

Retrieved records were exported from Scopus and Web of Science and processed in R. Duplicate detection was performed in two sequential steps. First, DOI fields were normalized by converting all characters to lowercase, removing DOI URL prefixes, trimming spaces, and standardizing punctuation. Exact DOI matches across Scopus and Web of Science were flagged as duplicates. Second, records without DOI or with inconsistent DOI information were compared using normalized titles. Title normalization included lowercasing, removal of punctuation, removal of diacritics, standardization of special characters, and conversion of Unicode variants such as VO_2_ to VO2. Potential duplicate records identified through title matching were manually checked before removal. When duplicate records were identified, the record with the most complete bibliographic metadata was retained. The integrity of the dataset was verified using publication year, title, author, source title, DOI, citation, and keyword fields.

Keyword harmonization was conducted before co-occurrence and thematic map analyses. All keywords were converted to lowercase, spelling variants were standardized, and synonymous terms were merged using a manually prepared thesaurus. For example, “VO2max,” “VO_2_max,” “maximal oxygen uptake,” and “maximal aerobic capacity” were reviewed and harmonized where conceptually equivalent. Similarly, threshold-related terms such as “ventilatory threshold,” “VT1,” “VT2,” “lactate threshold,” “LT1,” “LT2,” and “respiratory compensation point” were reviewed to preserve physiologically meaningful distinctions. Generic indexing terms that did not represent substantive concepts in sports physiology, such as “human,” “humans,” “article,” “male,” “female,” “adult,” and “controlled study,” were excluded from the primary conceptual interpretation to reduce indexing noise. The keyword thesaurus and stop-word list were retained as part of the analytical workflow.

### 2.6 Bibliometric indicators and analytical framework

In the study, descriptive, structural, conceptual, and social bibliometric indicators were used together. Trends in annual scientific production were examined, and the Compound Annual Growth Rate (CAGR) was calculated to determine the long-term growth rate of the field. The annual production trend was analyzed using simple linear regression model (*y* = β0 + β1x), where the dependent variable was defined as the annual number of publications and the independent variable was defined as time (year). The model's explanatory level was assessed using the R^2^ coefficient. The Bradford Law was applied to examine journal concentration, and the clustering of core journals in the literature was identified. Author productivity was analyzed within the framework of Lotka's Law to test whether or not production was concentrated in a specific group of authors.

The conceptual structure within the field was examined through keyword co-occurrence networks and thematic maps; strategic nodes within the network were identified using degree, betweenness, and closeness criteria. Conceptual and thematic analyses were conducted using the fields of keywords indexed in the databases (Scopus Index Keywords and Web of Science Keywords Plus). In thematic map and co-occurrence analyses, the minimum keyword frequency was set at 5. This prevented low-frequency terms from adding noise into the network structure while preserving the thematic diversity. Maps of international collaboration were drawn up to analyse the international distribution of scientific production. No minimum publication threshold was set for the country collaboration analyses and all countries in the dataset were included in the analysis. Additionally, the citation half-life was calculated based on the median reference year derived from the distribution of publication years of the references, and the time-based distribution of citation density was assessed using Q25–Q75 quartile values. In network analyses, the Association Strength normalization method was used to ensure the comparability of link strength and to limit the bias introduced by nodes with high production volumes (Van Eck and Waltman, 2014). The full counting approach was adopted in the analyses, and each co-authorship, country link and keyword co-occurrence were assessed with equal weight. All analyses were conducted in R version 4.5.2 using the bibliometrix package version 5.2.1. The complete analytical workflow, including data cleaning, duplicate removal, keyword harmonization, and visualization procedures, was preserved to ensure reproducibility. The bibliometric calculations and network-based analyses were done using the Bibliometrix 5.2.1 software. The time zone was set to Europe/Istanbul, and the reproducibility of the analysis process was ensured.

## 3 Results

In this study, the relevant literature from 2010 to 2025 was reviewed, and a total of 2,745 unique original publications were included in the bibliometric analysis after database filtering, merging, and strict duplicate removal.

Table 1 summarizes the dataset construction process. A total of 3,740 records were initially identified from Scopus and Web of Science Core Collection. After database filters were applied, 3,407 records remained. Following strict duplicate removal based on normalized DOI and title matching, 2,745 unique records were retained for bibliometric analysis. These figures indicate that the combined use of Scopus and Web of Science expanded the coverage of the field while duplicate removal ensured that the final corpus contained unique bibliographic records.

**Table 1:** The scope of the datasets and the number of records.

Set	*N*
Scopus records identified	2,061
Web of Science records identified	1,679
Total records identified	3,740
Scopus records after database filters	1,849
Web of Science records after database filters	1,558
Total records after database filters	3,407
Duplicate records removed	662
Final unique records included	2,745

**Figure 2:**
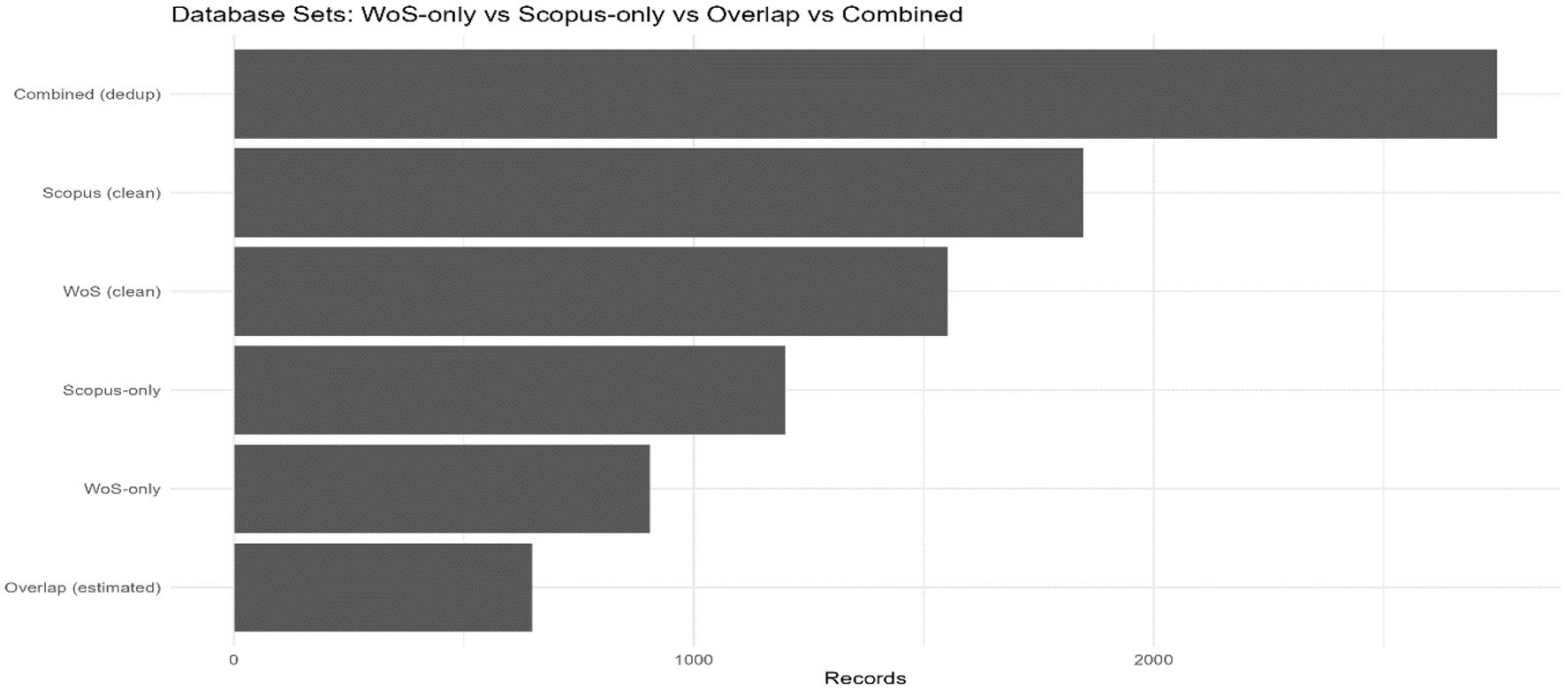
Distribution of publication numbers by databases.

The Venn diagram/bar chart in Figure 2 illustrates the overlap and unique coverage of the two databases after database filtering. The overlap between Scopus and Web of Science was 662 records, whereas 1,187 records were unique to Scopus and 896 records were unique to Web of Science. This distribution confirms that WoS and Scopus are not substitutes but complementary structures in this field of study, and the use of combined data is essential for a more holistic bibliometric analysis.

**Figure 3:**
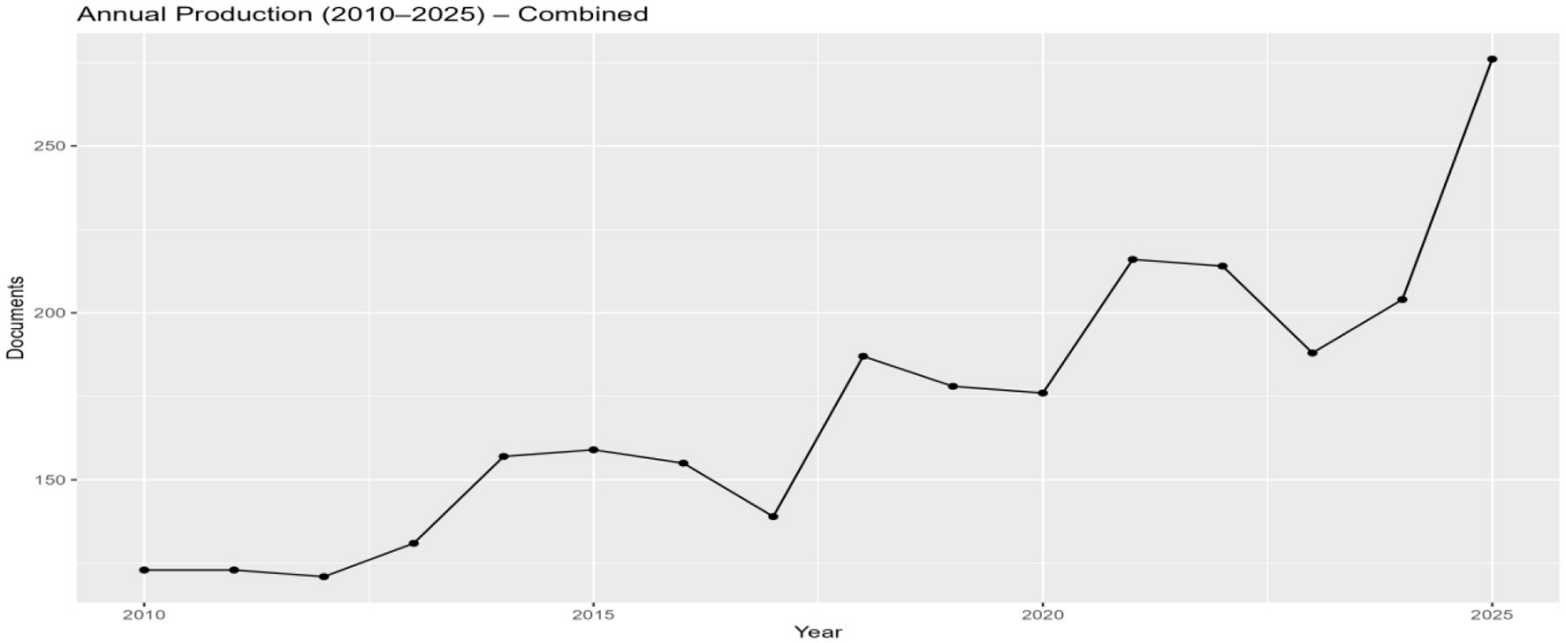
Publication production in the combined dataset by year.

Figure 3 shows the development of total scientific production between 2010 and 2025. The graph reveals that academic interest in the field has steadily grown over the last 15 years. In particular, the upward acceleration of the curve since 2018 indicates that the topic remains up-to-date and is gaining increased prominence in the sports sciences and physiology literature.

**Figure 4:**
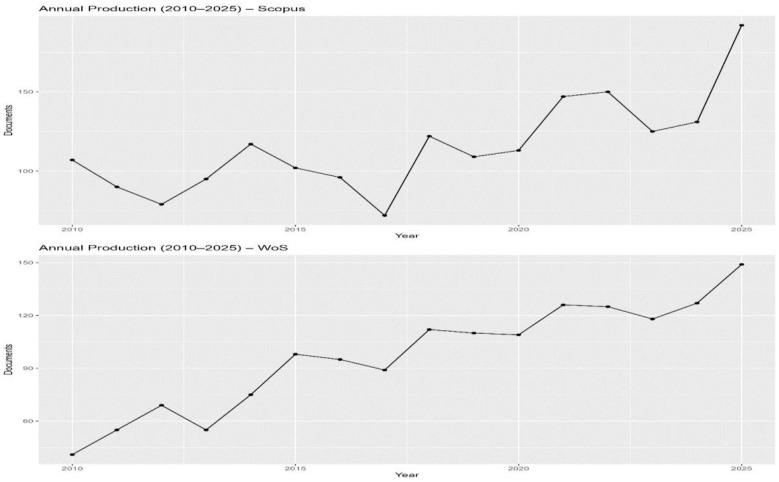
Comparison of annual publication production between WoS and scopus.

When comparing the annual production performance of the databases, it appears that the number of publications in Scopus is higher in volume compared to WoS (Figure 4). Nevertheless, the increase and decrease observed in both databases during similar years (parallel trend lines) indicate that the global growth trend in the field of research is confirmed by both indices.

The data for 2010–2025 indicated that the annual number of publications increased from 123 to 276. The calculated Compound Annual Growth Rate (CAGR) was 5.53%, indicating a steady growth in academic interest in the field (Table 2).

**Table 2:** Annual production and growth indicators: CAGR (2010–2025) = 5.536%.

PY	Documents
2010	123
2011	123
2012	121
2013	131
2014	157
2015	159
2016	155
2017	139
2018	187
2019	178
2020	175
2021	215
2022	214
2023	188
2024	204
2025	276

When examining Table 3, it was determined that the results of the regression analysis (R^2^ = 0.81) statistically confirmed that the growth trend shown in Table 2 had a strong and predictable growth pattern rather than random fluctuations.

Table 3Trend regression coefficients and model fit.

**Table:** 

*N*	*R2*	AdjR2	*F*	df1	df2	CAGR_percent
16	0.8094838	0.7958755	59.48456	1	14	5.536

**Table:** 

Term	Estimate	Std Error	*t*	*p*
(Intercept)	−15,953.602941	2,090.755209	−7.630546	0.000002364094
PY	7.992647	1.036307	7.712623	0.000002090987

When examining Table 4, it was observed that the literature on the concepts of cardiopulmonary performance and threshold in a sports context, as analyzed using Bradford's Law, had a distinct core-journal structure. Accordingly, while only seven journals accounted for 31.3% of total publications, the 44 journals in the second zone constituted 35.2% of publications. On the other hand, the publications by 531 journals represented 33.5% of the total literature in the third zone. This distribution revealed that the field was concentrated around a limited number of high-impact core journals, while also demonstrating a trend toward interdisciplinary expansion. The relatively balanced publication rates across the zones indicated that the relevant research field possessed both a mature and sustainable scientific structure.

**Table 4:** Distribution of journals and publications by bradford zones.

Zone	Journals	Articles	Share_articles
Zone 1	7	858	0.313
Zone 2	44	967	0.352
Zone 3	531	920	0.335

**Figure 5:**
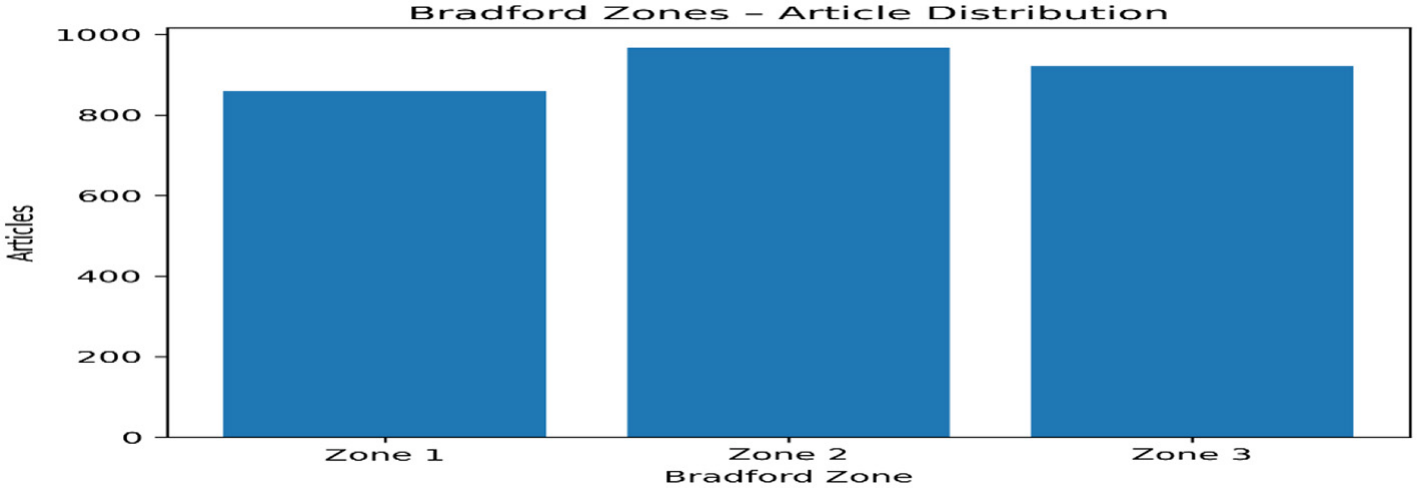
Distribution of the publications by bradford zones.

Figure 5 shows that the literature has a core-periphery structure according to Bradford's Law. The production of just seven journals (Zone 1), accounting for 31.3% of all publications, demonstrates that the field has a strong core publication structure. This indicates that cardiopulmonary performance and threshold-based studies are concentrated around certain high-impact journals. The share of publications by the 44 journals in Zone 2, accounting for 35.2% of publications, demonstrates that the field is not limited to central journals alone, but rather forms an interdisciplinary production area expanding around a core. On the other hand, the production of 531 journals listed in Zone 3, accounting for 33.5% of all publications, demonstrates that the literature is disseminated across a broad publishing ecosystem, yet production has been still driven by central hubs. The balanced distribution across the zones indicates that the field has both a mature and sustainable structure.

**Figure 6:**
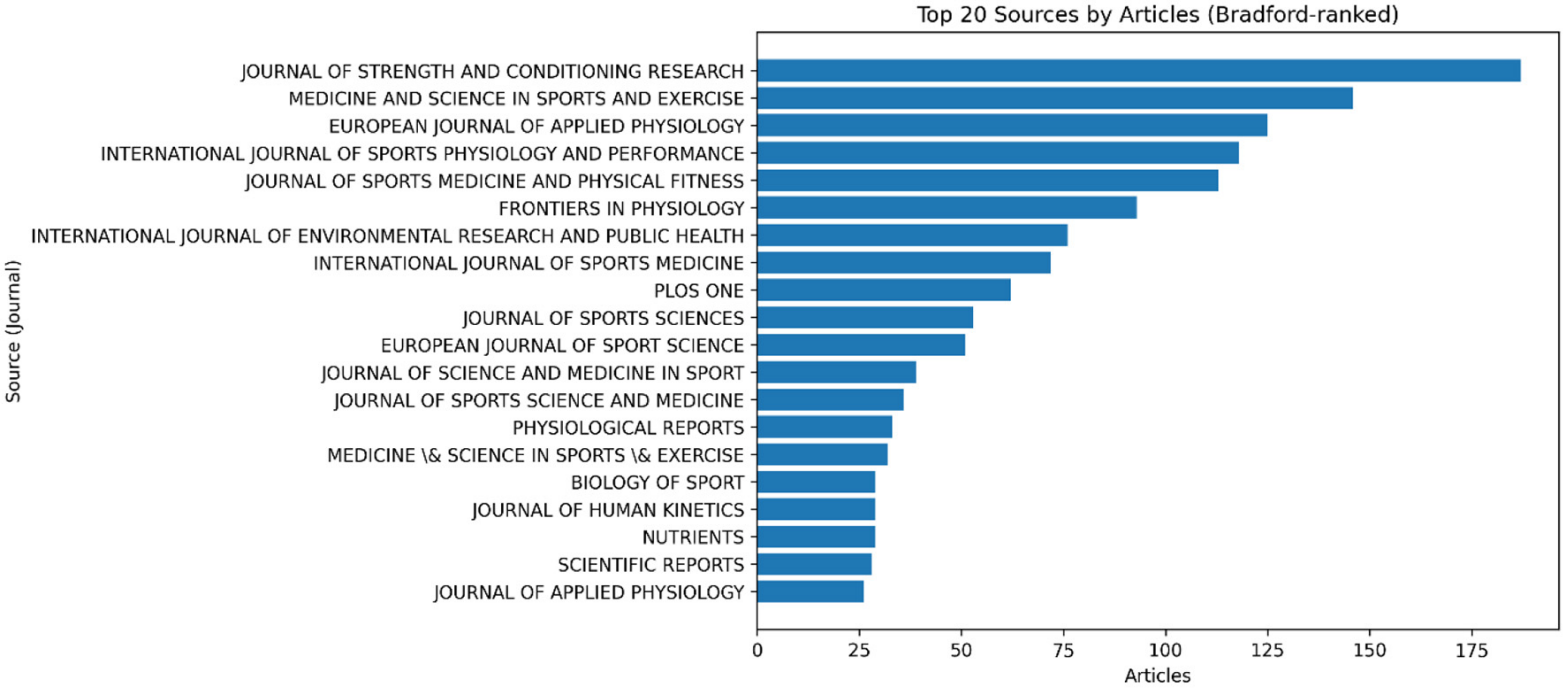
The 20 most productive journals (bradford ranking).

Figure 6 illustrates the core journals that determine the publication intensity of the literature. As is seen, the top 20 most productive journals play a decisive role in the total publication. The specialization of the vast majority of these journals in the fields of sports physiology, exercise science and practical performance indicates that the research field is structured around a theoretical and practical performance axis. This finding demonstrates that the literature is not randomly distributed but is guided by specific epistemic centers and reveals that the field has an institutionalized publication structure.

**Figure 7:**
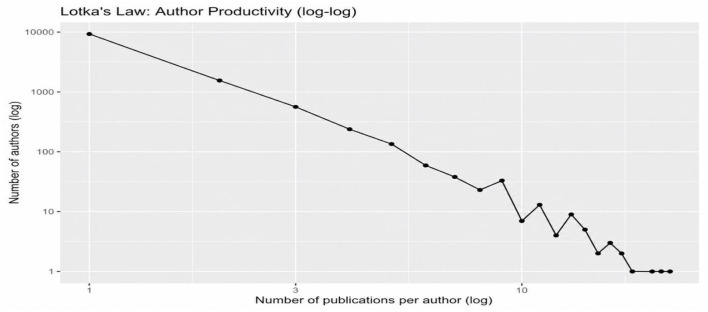
Authors' productivity according to Lotka's law (log–log).

The log–log distribution plot presented in Figure 7 shows that the literature exhibits a productivity model consistent with the classical Lotka's law. A small number of authors produce a high number of publications, while the vast majority make only a limited number of contributions.

The production of the most productive authors, as shown in Table 5, with 15–22 publications, indicates that the field is concentrated around specific research groups. This highlights that leading research centers and long-term specialization are decisive factors in research on cardiopulmonary performance. The distinctiveness of the Lotka distribution indicates that the field possesses an epistemically consolidated structure and knowledge production is sustained through specific research networks.

**Table 5:** The top 10 most productive authors.

AU	Publications
Murias J	22
Guglielmo L	21
Sperlich B	20
Zagatto A	18
Meyer T	17
Wahl P	17
De L R	16
Millet G	16
Wiecha S	16
Bishop D	15

**Figure 8:**
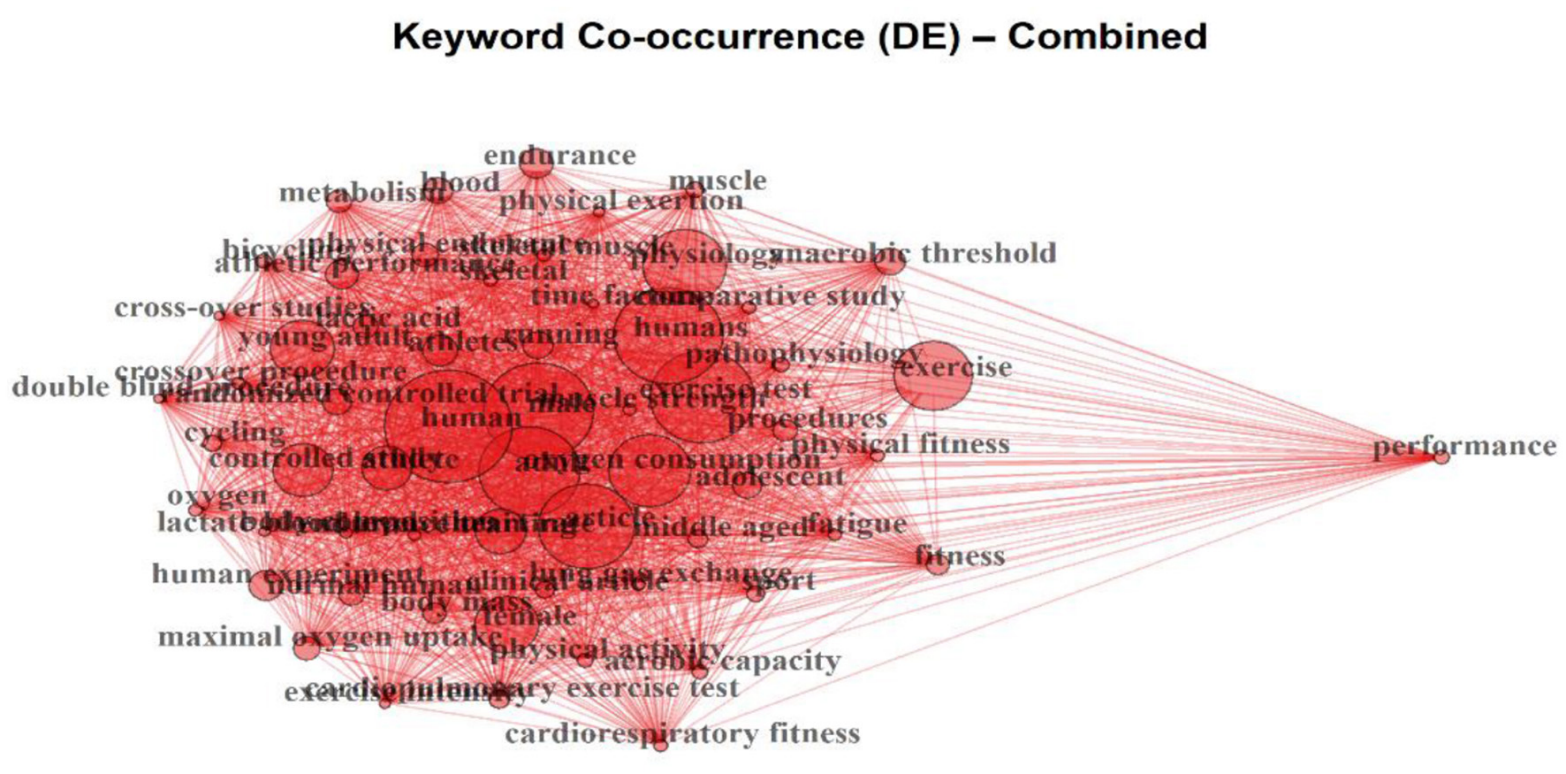
Keyword co-occurrence network (combined dataset).

The keyword co-occurrence network presented in Figure 8 demonstrates that the conceptual structure of the literature is concentrated around human-centered exercise physiology. The high connectivity of the concepts “human”, “exercise”, “oxygen consumption”, and “anaerobic threshold” at the center of the network highlights that physiological thresholds and oxygen metabolism are key determinants in research on cardiopulmonary performance. The relatively decentralized yet strongly connected nature of the term “performance” to the core indicates that the concept of performance is treated in the literature not as an independent theme but as a result of physiological parameters. Furthermore, the prominent presence of methodological terms (such as “controlled” and “crossover” designs) within the network indicates that the field has been shaped by experimental studies conducted to high methodological standards. On the other hand, the formation of distinct sub-clusters by sport-specific terms (cycling and running) shows that the literature is diversified contextually for practical sports performance.

**Figure 9:**
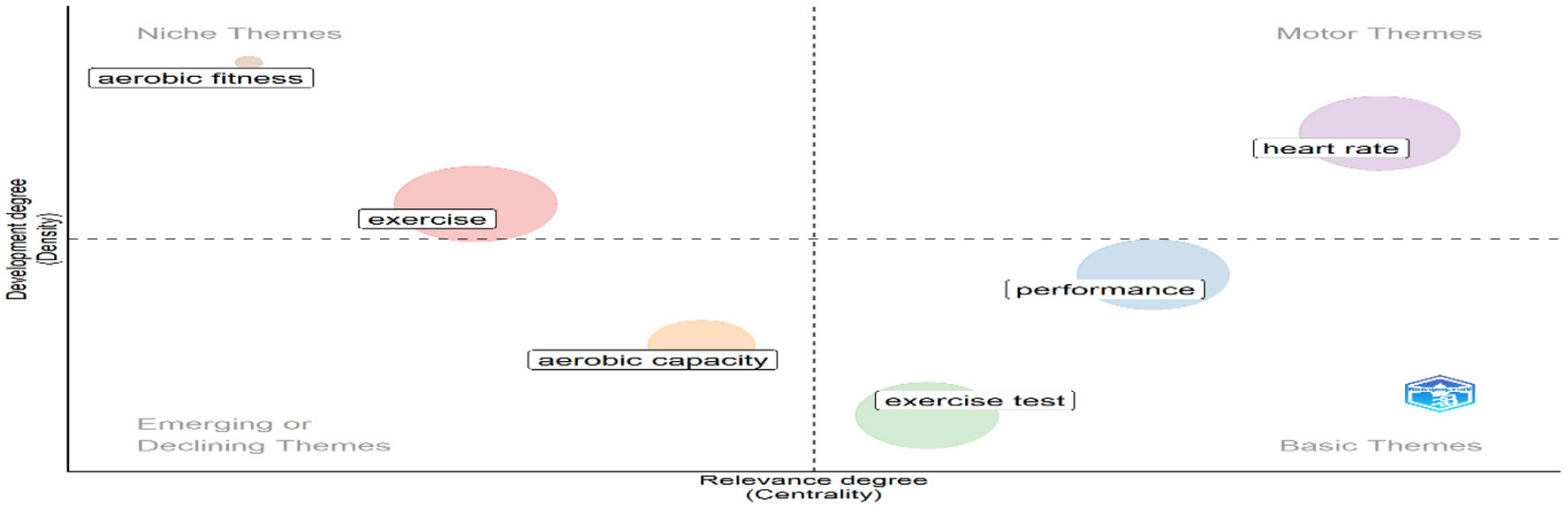
Thematic map based on keywords (quadrants).

Based on Figure 9, the inclusion of VO_2_max and exercise performance themes in the Motor Themes region (high centrality–high density) indicates that these concepts are both advanced and central components of the field. The presence of concepts “ventilatory threshold” and “lactate threshold” in the Basic Themes region indicates that these themes are the fundamental building blocks of the field; however, their conceptual density is less than that of motor themes. The inclusion of more specific topics, such as isocapnic buffering in the Niche Themes region, suggests that these topics are studied by specialized but relatively narrow research clusters. On the other hand, the themes in the Emerging/Declining region reflect the field's evolutionary dynamics. This structure demonstrates that the literature has both stable core themes and emerging sub-fields.

**Figure 10:**
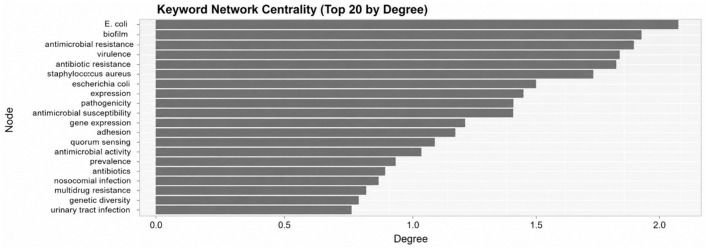
Keyword network based on degree.

Upon examination of Table 6 and Figure 10, the centrality analysis indicates the strongest nodes within the conceptual network are HUMAN, EXERCISE, OXYGEN CONSUMPTION, and ANAEROBIC THRESHOLD. In particular, the high betweenness value of the term “EXERCISE” shows that this concept acts as a bridge between different thematic clusters. This suggests that research on cardiopulmonary performance has a structure that links the axes of performance, physiology, and clinical practice. The high centrality values of the term “anaerobic threshold” indicate that threshold-based physiological parameters hold a critical position in the literature. This finding corroborates the conceptual framework of the study.

**Table 6:** The top 20 keywords with the highest centrality.

Node	Degree	Betweenness	Closeness
Human	4.366	687.256.12	0.00008483203
Article	4.072	569.709.12	0.00008231808
Male	4.011	533.635.88	0.00008150624
Exercise	3.890	2.283.231.87	0.00008587377
Humans	3.865	841.923.92	0.00008534608
Adult	3.836	520.030.34	0.00008204792
Exercise test	3.658	593.242.71	0.00008202100
Oxygen consumption	3.114	383.486.19	0.00007794232
Physiology	3.039	528.809.59	0.00008082768
Female	2.989	414.691.32	0.00007903889
Controlled study	2.984	327.894.46	0.00007727975
Young adult	2.589	257.869.24	0.00007559722
Athlete	2.505	241.548.03	0.00007514842
Heart rate	2.418	254.940.88	0.00007589557
Human experiment	2.130	207.704.29	0.00007538067
Maximal oxygen uptake	2.048	217.673.03	0.00007428869
Endurance	2.007	516.556.28	0.00007751337
Metabolism	1.821	402.427.20	0.00007648184
Anaerobic threshold	1.799	523.592.37	0.00007627183
Athletes	1.771	126.150.71	0.00007195798

**Figure 11:**
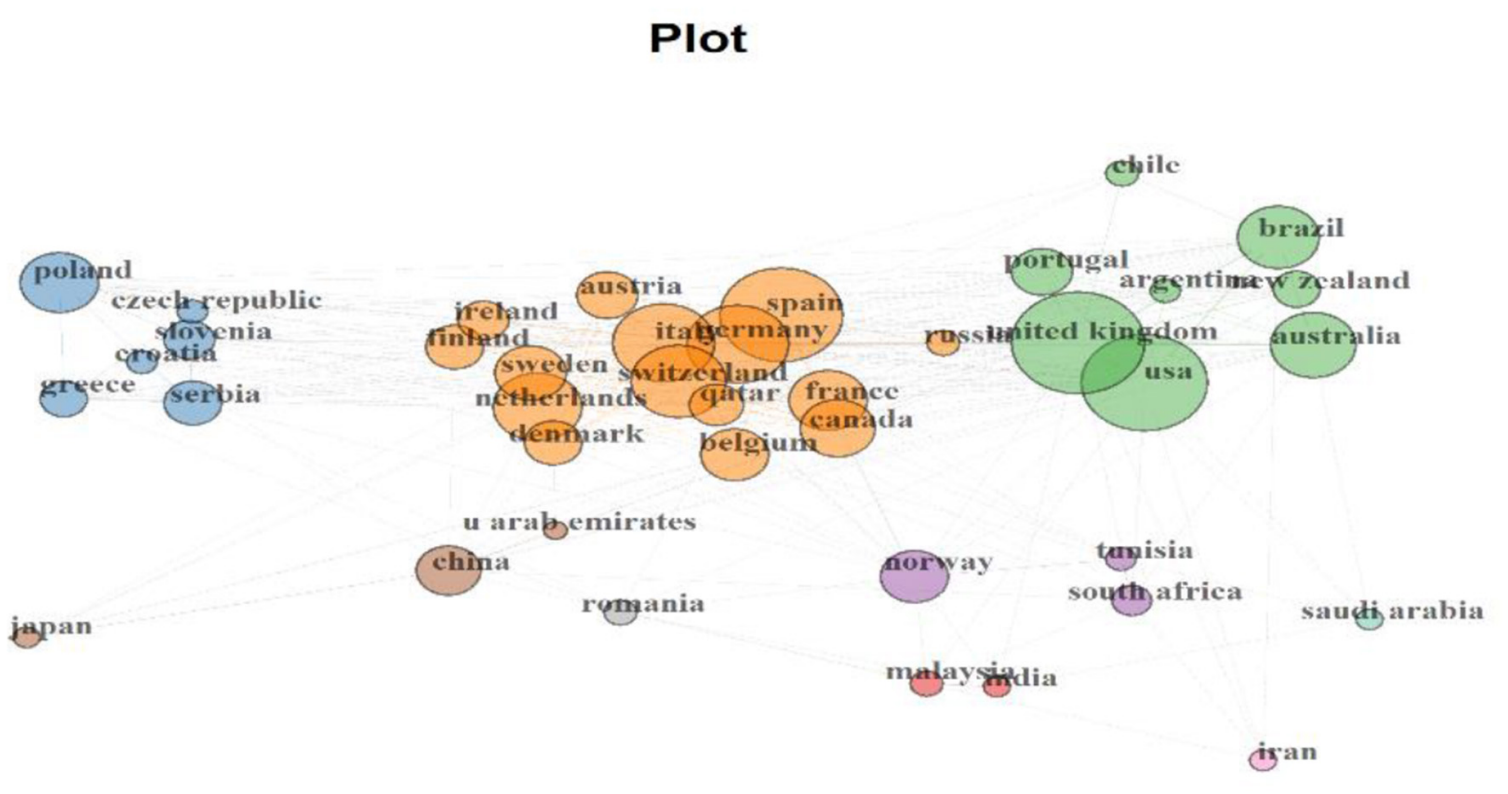
Country collaboration network.

Based on Figure 11, the analysis of the country collaboration network indicates that the literature has an intense collaboration structure at the international scale. The central position of the USA and European countries within the network indicates that these countries play a leading role in knowledge production within the field. The network's high node density and connectivity level demonstrates that research on cardiopulmonary performance has been conducted within a wide network and that interdisciplinary collaboration is highly structured.

**Figure 12:**
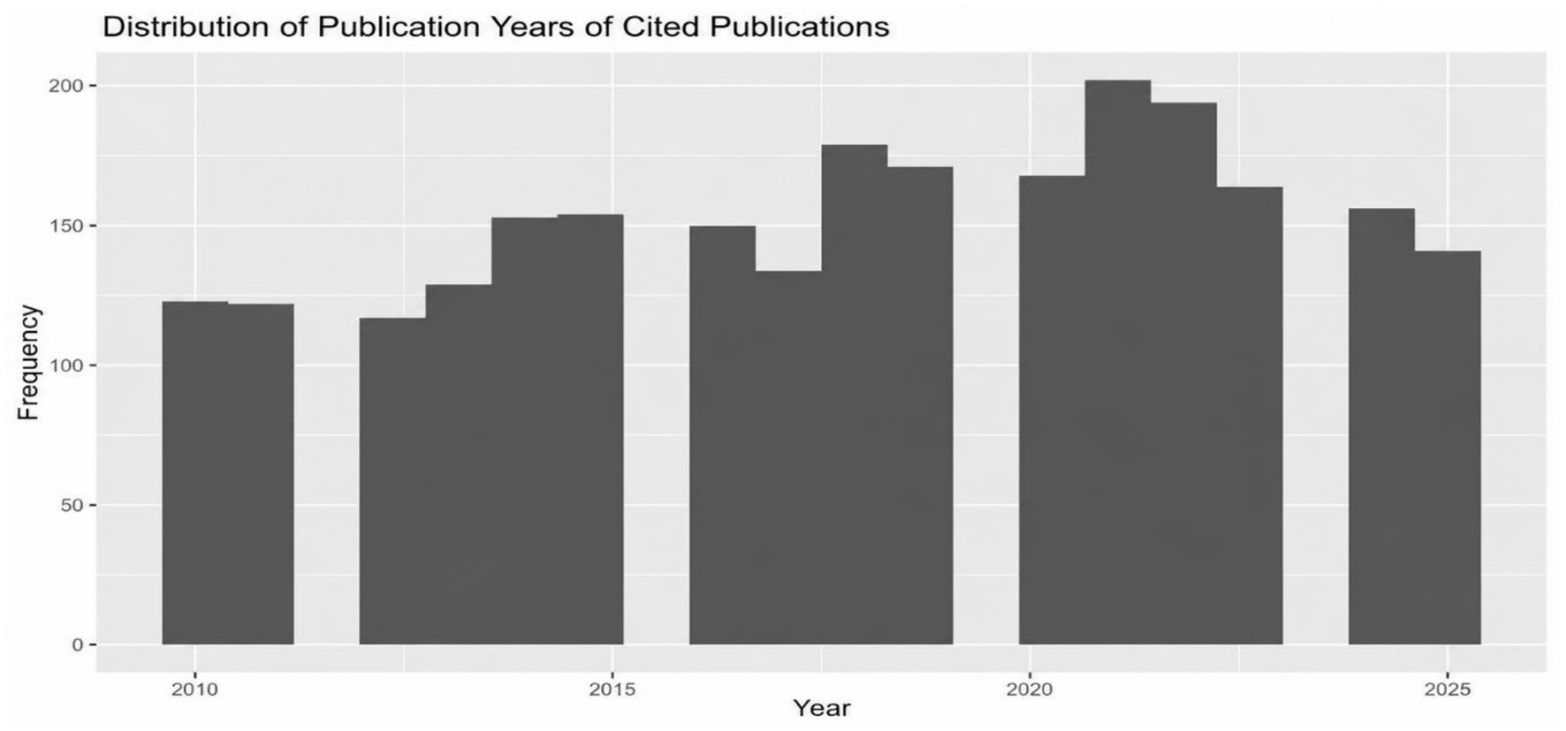
Yearly distribution of cited publications.

Referring to Table 7 and Figure 12, the median reference year was calculated as 2018 based on the citation half-life analysis. This indicates that the literature refers to relatively recent studies. The Q25 and Q75 values (for the 2014–2022 period) reveal that the citation distribution is balanced and medium-term. This result demonstrates that the field has a current and continuous knowledge structure. The lack of a clear concentration of citations in a single period suggests that the literature has undergone evolutionary development rather than sudden paradigm shifts.

**Table 7:** Citation half-life statistics.

*N*_total	*N*_cited	HalfLife_ MedianYear	Q25	Median	Q75
2.745	2.457	2.018	2.014	2.018	2.022

## 4 Discussion

This study aimed to reveal the global evolution, production dynamics, structural concentration and conceptual patterns of research on cardiopulmonary performance and physiological threshold in the field of sports sciences during 2010–2025 through a holistic approach and within a bibliometric framework.

An analysis of the findings presented in Table 1 and Figure 2 shows that there is only an overlap of 23.6% between the Web of Science and Scopus databases, and 76.4% of the literature is found in individual indices. This finding points out that in certain bibliometric studies, the selection of the database can have a direct and decisive impact on the results (Mongeon and Paul-Hus, 2016). On the other hand, it is reported that the Web of Science database has historically followed a more selective and core-journal-focused indexing strategy, whilst Scopus has broader journal coverage (Falagas et al., 2008). It is stated that these structural differences can lead to representational differences, particularly in interdisciplinary fields such as sports sciences, which cover clinical physiology, practical performance, and exercise-based studies (Archambault et al., 2009). Furthermore, the low overlap rate suggests that the literature on cardiopulmonary performance is divided between central and peripheral publication ecosystems (Leydesdorff and Wagner, 2008). However, it has also been demonstrated that analyses based on a single database have the risk of systematically under-representing the volume of publication production and citation networks (Harzing and Alakangas, 2016). Consequently, the hybrid integration approach adopted in the present study is considered a methodological preference aimed at more accurately representing the conceptual and structural integrity of the field (Martín-Martín et al., 2018). According to these results, research on cardiopulmonary performance and threshold is characterized by a multi-center, interdisciplinary, and dispersed publication structure (Birkle et al., 2020).

The annual publication production graph presented in Figure 3 indicates that research on cardiopulmonary performance and physiological threshold has grown steadily between 2010 and 2025. The rise in annual publication production from 123 in 2010 to 276 in 2025, with a compound annual growth rate (CAGR) of 5.53%, indicates that the field has been going through a long-term and sustainable expansion process.

Some studies have indicated that the growth dynamics of scientific fields generally exhibit non-linear, clustered structures characterized by periodic leaps (Price, 1963). Furthermore, it has been noted that modern scientific publication production has been expanding at a steady yet discipline-specific rate on a global scale, with annual growth rates ranging between 4% and 8% in most disciplines (Bornmann and Mutz, 2015). Another study has reported that scientific fields expand in a scalable manner over time in terms of network density and volume of knowledge production, and this growth has a structural characteristic (Fortunato et al., 2018). In this regard, the CAGR value (5.53%) calculated in this study shows that the literature on cardiopulmonary performance within the context of sports sciences exhibits a stable expansion model consistent with global scientific growth trends.

The high explanatory power of the regression model reported in Table 3 (*R*^2^ = 0.81) reveals that this growth is based on a systematic production trend rather than random fluctuations.

The acceleration observed after 2018 should not be interpreted merely as a database coverage effect. It likely reflects the convergence of several developments in sports physiology: the growing use of threshold-based training-intensity distribution models, the increasing availability of field-oriented physiological monitoring tools, and the methodological shift from single maximal indicators toward individualized intensity domains. Contemporary endurance training models commonly define zones using VT1/LT1 and VT2/LT2 or RCP boundaries, which has increased the visibility of threshold-related concepts in both laboratory and applied sport settings. In parallel, wearable and non-invasive approaches, including sweat lactate sensors and HRV-derived threshold markers, have expanded the methodological vocabulary of the field and may partly explain the emergence of new keyword clusters related to real-time monitoring and automated threshold detection (Gronwald et al., 2020; Van Hoovels et al., 2021; Seki et al., 2021; Casado et al., 2022; Muramoto et al., 2023; Tanner et al., 2024).

The parallel growth curves exhibited by Web of Science and Scopus data in Figure 3 indicate that this growth is not index-specific but rather reflects the general dynamics of the field. This emerging parallelism has been reported to reflect that research on cardiopulmonary performance takes place in a globally expanding scientific network and that the growth in production reflects a structural paradigm expansion (Wagner and Leydesdorff, 2005).

The zone distribution presented in Table 4 and Figure 5 reveals that the literature on cardiopulmonary performance and physiological threshold has a distinct core-periphery publication structure. Furthermore, the production of just seven journals accounting for 31.3% of the total publications indicates that knowledge has become institutionalized on a limited number of high-impact publication platforms.

The Bradford's Law stipulates that the distribution of scientific knowledge exhibits an unequal and concentrated pattern and states that this concentration is a structural indicator of a field's maturation (Bradford, 1985). However, modern bibliometric analyses have shown that the clustering of core journals is strongly correlated with the level of institutionalization within research fields (Egghe and Rousseau, 2003). Such concentrations observed in scientific publication production indicate that knowledge production is shaped around specific central publication platforms and specialized research groups and the level of specialization within the field has been rising (Glanzel, 2003).

The production of the 44 journals listed in Zone 2, accounting for 35.2% of all publications, indicates that the field has a publication structure that expands around a core and involves interdisciplinary interaction. Therefore, such semi-centralized zone structures point to the simultaneous existence of both specialized and applied publication platforms in mature research fields (Waltman, 2016).

The production share of 33.5% held by the 531 journals in Zone 3 indicates that the literature is disseminated across a broad publication ecosystem, yet the intensity of publication production is still determined by core centers. A study supporting this finding reported that the correlation between journal concentration and field size is particularly more pronounced in disciplines such as performance science, which involve both clinical and applied components (Mingers and Leydesdorff, 2015). On the other hand, examination of Figure 5 reveals that the pronounced production density of the top 20 most productive journals indicates that research on cardiopulmonary performance has attained an institutionalized and methodologically standardized core knowledge structure.

Based on Figure 7, the keyword co-occurrence network indicates that the literature on cardiopulmonary performance is structured around a conceptual framework based on physiological threshold. Furthermore, the high connectivity of the concepts “*exercise”, “oxygen consumption” and “anaerobic threshold,”* which are centered in the network, demonstrates that the field is organized around physiological determinants rather than performance outcomes.

Some studies in the literature indicate that oxygen consumption and threshold parameters play the most fundamental role in determining endurance performance (Joyner and Coyle, 2008). On the other hand, it has been stated that the performance power and VO_2_max, which are frequently used in sports performance, explain endurance capacity separately from the ventilatory and lactate thresholds (Faude et al., 2009). In this sense, the presence of physiological concepts at the center of the network suggests that the literature is structured along the line of mechanistic physiology rather than practical performance research. Furthermore, the strong links between the term “performance” and core physiological nodes indicate that in the field of sports science, performance is not treated as an independent concept but rather as an outcome of physiological capacity. It has been reported that this structure is compatible with the structure in the literature that explains performance analysis in sports physiology through biological systems (Poole and Jones, 2012).

The thematic map analysis in Figure 9 reveals that the literature on cardiopulmonary performance exhibits a balanced structural distribution centered around both advanced and fundamental themes.

Motor themes with high centrality and density values in the thematic maps represent the conceptual cores of the field, indicating its maturity and guiding the direction of research (Callon et al., 1991). Furthermore, the positioning of the concepts VO_2_max and exercise performance within the motor themes region indicates that endurance performance is defined through physiological determinants. In other words, studies dating back many years have determined that VO_2_max represents the upper limit of aerobic capacity and is one of the most fundamental determining parameters of sporting performance (Bassett, 2000). Furthermore, it has been reported that performance is related not only to maximal capacity but also to threshold-based sustainable parameters (Joyner and Coyle, 2008).

In particular, when the maps are examined, the placement of the concepts “ventilatory threshold” and “lactate threshold” within the basic themes region indicates that these parameters form the theoretical infrastructure of the literature; however, when considered from as study diversity, they are more limited compared to motor themes.

It has been reported that the concepts of lactate and ventilatory thresholds are highly sensitive in predicting endurance performance; however, methodological standards have become more stable over the years (Faude et al.,2009). The position of lactate threshold and ventilatory threshold as basic themes can be interpreted as evidence of conceptual stabilization rather than limited importance. These concepts are used across multiple subfields, including running, cycling, swimming, training prescription, fatigue modeling, and performance diagnostics. Therefore, they show high centrality because they function as shared methodological anchors across the field. However, their lower thematic density compared with motor themes suggests that threshold concepts are distributed across several applied and physiological contexts rather than concentrated in a single internally specialized cluster. This interpretation is consistent with the continuing methodological heterogeneity surrounding threshold determination, including visual inspection, fixed lactate concentrations, Dmax-derived thresholds, maximal lactate steady state, LT1/LT2 terminology, and VT1/VT2 or RCP-based gas-exchange approaches (MacIntosh et al., 2021; Kim et al., 2021; Tanner et al., 2024).

The isocapnic buffering phase, a deterministic concept in performance parameters yet typically discussed in limited themes and clusters, indicates that these parameters are associated with lines of research that examine more specific metabolic mechanisms. It has been reported that the isocapnic buffering phase is a physiological transition process between the ventilatory threshold and the respiratory compensation point, demonstrating the effectiveness of the bicarbonate buffer system against metabolic acidosis. From this perspective, it has been stated that the isocapnic buffering phase is associated with acid-base balance and ventilatory compensation processes during exercise and generally requires advanced laboratory analyses (Wasserman et al., 2012). Therefore, this theme appears to be concentrated within specialized physiological sub-research clusters rather than the broader performance literature.

The observation of such structures in thematic maps is assessed as a typical pattern indicating that the field possesses both maturity and potential for evolutionary expansion (Cobo et al., 2011).

A further explanation for the observed keyword structure is the current debate on threshold detection methods. Traditional ventilatory threshold and respiratory compensation point determination often relies on expert visual inspection of gas-exchange plots, but this approach may be affected by evaluator experience, inter-observer variability, and protocol characteristics. Recent studies have therefore proposed computerized or algorithmic approaches to improve reproducibility and standardization. Kim et al. (2021), for example, developed an automated method for determining ventilatory threshold and respiratory compensation point, while HRV-based DFA-alpha1 approaches have been proposed as practical alternatives for estimating exercise intensity boundaries outside conventional laboratory settings. These developments help explain why threshold-related terms remain central while newer methodological terms appear as emerging or niche themes (Gronwald et al., 2020; Kim et al., 2021; Tanner et al., 2024).

The coexistence of terms such as anaerobic threshold, lactate threshold, ventilatory threshold, LT1, LT2, VT1, VT2, and RCP also reflects unresolved nomenclature issues in the field. The term “anaerobic threshold” has historically been used to refer to different physiological breakpoints, sometimes corresponding to the first threshold and sometimes to the second threshold. For this reason, recent literature increasingly recommends specifying both the measurement domain and the threshold level, such as LT1/LT2 for lactate-derived thresholds and VT1/VT2 or RCP for gas-exchange-derived thresholds. In the present bibliometric map, the coexistence of these terms likely reflects both conceptual continuity and terminological heterogeneity within sports physiology (MacIntosh et al., 2021; Mazaheri et al., 2021).

The concepts placed in the niche themes region of the thematic map represent research lines that are highly dense internally in the literature but exhibit more limited connections in terms of the basic degree of the network (Aria and Cuccurullo, 2017). In this sense, the literature on isocapnic buffering is not an overarching concept guiding the broader performance paradigm but rather serves as a specific line of research capable of explaining the lower-level regulatory mechanisms of threshold-based physiological models.

When such thematic positions are examined, they reflect a trend toward vertical deepening from core concepts to more detailed mechanistic subtitles as the field matures (Donthu et al., 2021). The niche position of isocapnic buffering indicates that the literature has expanded toward lower-level metabolic control mechanisms. The consistency of this conceptual distribution with the network structure has been assessed through centrality metrics. The degree, betweenness, and closeness values of the concepts demonstrate the consistency of the core and niche positions observed on the thematic map with the structural network dynamics and ensure that the conceptual organization of the literature is supported by quantitative network indicators.

Examination of the centrality analyses in Figure 10 and Table 6 reveals the conceptual organization of the literature on cardiopulmonary performance through quantitative network indicators. After accounting for generic indexing terms, the substantive conceptual core of the keyword network was structured around exercise, oxygen consumption, maximal oxygen uptake, endurance, heart rate, and threshold-related parameters.

The central position of exercise and oxygen consumption indicates that the literature is organized around physiological determinants of performance rather than performance outcomes alone. In particular, the role of maximal oxygen consumption and threshold parameters in explaining performance capacity is highlighted by some studies in the latest literature (Jones and Vanhatalo, 2017; Poole et al., 2016).

The high betweenness value of the concept “EXERCISE” for the betweenness degree reveals that this term acts as a bridge between different thematic clusters. In network analysis, the betweenness degree is used to identify the checkpoints of information flow and the connections between concepts with strategic nodes (Borgatti et al., 2024; Van Eck and Waltman, 2014). Based on this information, the concept “exercise” supports the interdisciplinary nature of the field by forming a methodological interface between physiological threshold models and performance outcomes.

Concepts with high closeness are located at shorter distances to other nodes of the network on average, indicating that these concepts hold a central position for the accessibility of information. However, the accessibility of exercise and human-based physiological variables in the field of cardiopulmonary performance suggests that the field is organized around the axis of experimental human physiology. Therefore, when the degree metrics are assessed together, this shows that the motor and basic themes observed on the thematic map are supported around the network structure. This result suggests that the conceptual core of cardiopulmonary performance research has consolidated both thematically and structurally around physiological threshold parameters and exercise-based mechanisms.

On the other hand, when Figure 11 is analyzed, the country collaboration network shows that cardiopulmonary performance and physiological threshold research are concentrated around certain central countries at the global level. Moreover, countries with high link density in the network structure constitute the structural core of the international collaboration network.

Bibliometric studies have indicated that the centrality outputs of country-level collaboration networks provide an important structure in explaining the global distribution of scientific publication production and the geographical orientation of knowledge flows (Wagner and Leydesdorff, 2005; Van Eck and Waltman, 2014). Countries where international co-authorship networks are concentrated generally represent scientific claims with high research infrastructure, advanced performance laboratories and strong funding mechanisms. Research on global science networks emphasizes that countries with high degrees have a guiding role in knowledge production and are decisive in shaping the research agenda (Leydesdorff and Wagner, 2008).

Network analyses show that not only has the number of international co-publications risen, but also the network density and the quality of contextual connection have changed over time. Recent scientometrics analyses have reported that global collaboration networks have evolved into a more complex and polycentric structure, revealing both powerful actors at the center and link dynamics with peripheral countries (Ronda-Pupo, 2024). In this sense, the country collaboration network reveals that the field of cardiopulmonary performance is organized within a globalized scientific system and exhibits hierarchical but diffuse network relationships around specific scientific actors. Such global network structures expand the boundaries of knowledge production by strengthening interdisciplinary collaboration.

The citation life analyses in Table 7 and Figure 12 demonstrate the dynamics of information obsolescence in the cardiopulmonary performance literature. The calculation of the median reference year as 2018 shows that the studies cited in the literature are concentrated in a relatively recent time period (Bornmann and Daniel, 2008; Wang et al., 2013).

Moreover, when Q25 (2014) and Q75 (2022) values are analyzed, it appears that the citation distribution is concentrated within an 8-year band. This suggests that the field is not only oriented toward the most recent work but also continues to take the core literature produced in the last decade as a reference (Wang et al., 2013). Moreover, an approximately 6–8-year citation cycle of cardiopulmonary performance and physiological threshold studies suggests that the field has a current and continuous scientific structure.

On the other hand, the lack of concentration of the year distribution of the cited publications in Figure 12 in a single period shows that the literature follows an evolutionary development model rather than sudden paradigm shifts. Such distributions in the scientific literature indicate that the field has gone through a process of steady growth and consolidation of knowledge (Fortunato et al., 2018). Thus, citation half-life analysis reveals that research on cardiopulmonary performance is both open to current knowledge production and systematically take the methodological and physiological foundational studies of the previous decade as a reference.

This study makes an original contribution to the literature by analyzing the cardiopulmonary performance and physiological threshold literature not only through production volume and citation indicators, but also in a holistic bibliometric framework in the context of conceptual structure, network centrality and international collaboration dynamics. While many studies in the field of sports sciences have focused on specific performance parameters or specific physiological models, this study systematically exposes the structural organization and information architecture of the field (Donthu et al., 2021).

Previous scientometric studies were generally conducted on a single database, and due to the limitation of data coverage, it is not possible to analyse the cognometric data (Mongeon and Paul-Hus, 2016). The hybrid data integration approach adopted in this study created a more realistic information map of the field by covering both the core and peripheral publication ecosystems of the literature. As such, the study provides an expanded frame of reference in the sports physiology literature with respect to methodological scope. Conceptual network and centrality analyses quantitatively demonstrated that endurance performance is consolidated around physiological threshold parameters. This finding suggests that the mechanistic foundations of performance physiology are both thematically and structurally central to the literature. The evaluation of network analysis together with the conceptual map shows that knowledge production in sport sciences is shaped not only by thematic density but also by structural connectivity (Van Eck and Waltman, 2014). Furthermore, citation half-life analyses revealed that the field follows an evolutionary and cumulative development model rather than sudden paradigm shifts. It has been reported that in the maturation processes of scientific fields, knowledge production consolidates around core concepts and then expands toward lower-level mechanistic topics (Fortunato et al., 2018). This study shows that the cardiopulmonary performance literature follows a similar structural evolution. Therefore, this research reveals that the field of cardiopulmonary performance in sport sciences is not only a growing literature but also a structurally consolidated, conceptually centralized, and globally organized scientific field. As such, the study makes a theoretical and methodological contribution to the literature in both applying bibliometric methodology and mapping the information architecture of performance physiology.

The findings of this study show that cardiopulmonary performance and physiological threshold research have important implications for applied sport sciences. Conceptual network and centrality analyses revealed that parameters based on exercise and oxygen consumption occupy a structural core position in the literature. This suggests that the current use of maximal oxygen consumption, ventilatory threshold, and anaerobic threshold measures as the main assessment tools in performance laboratories corroborates the scientific literature (Jones and Vanhatalo, 2017; Poole et al., 2016).

The thematic map results indicated that threshold parameters were located between motor and basic themes. This finding supports that endurance performance should be assessed not only through VO_2_max but also through sustainable metabolic capacity and threshold-based regulation mechanisms (Faude et al., 2009). Therefore, the use of loading zones based on ventilatory and lactate threshold in training planning offers an approach that aligns with the structural trends in the literature.

The positioning of the isocapnic buffering phase among niche themes suggests that this parameter is potentially a field for further investigation in advanced performance evaluations. The implementation of more detailed gas analysis protocols in performance laboratories may strengthen future assessments, especially considering the role of ventilatory compensation processes in determining high-intensity exercise tolerance in elite athletes (Poole et al., 2016).

The observed trend of centralization in the country collaboration network suggests that international partnerships in sports science research enhance information visibility and improve methodological standardization. This suggests that promoting multicentre models of data collection and international collaboration in sport performance research may boost scientific impact (Krawczyk et al., 2024). Finally, citation half-life analyses revealed that the literature has both a current and cumulative knowledge structure. This indicates that both classical physiological models and current methodological approaches should be evaluated collectively in practices of training science. Therefore, this study offers a scientific framework for decision-making processes for researchers in sports physiology, performance analysts and practitioners who work with elite athletes by revealing around which parameters the literature is consolidated.

The available findings suggest that the cardiopulmonary performance literature is consolidated around a physiological threshold-based core structure, but with potential for expansion in specific sub-themes. In particular, niche fields such as isocapnic buffering and ventilatory compensation processes have significant research potential in terms of advanced experimental designs and longitudinal studies in elite athlete populations (Poole et al., 2016; Jones and Vanhatalo, 2017). However, the integration of digital performance monitoring systems and data-driven analysis approaches into sports physiology may contribute to the emergence of novel conceptual clusters in the literature in the future (Fortunato et al., 2018). From a bibliometric point of view, the continuation of studies based on the integration of multiple databases will contribute to a more holistic presentation of the conceptual and structural maps of the field (Mongeon and Paul-Hus, 2016).

## 5 Limitations

This study has several limitations. First, the analysis was based only on records indexed in Web of Science Core Collection and Scopus; therefore, relevant publications indexed exclusively in other databases may have been missed. Second, only English-language article-type records were included, which may have introduced language and document-type bias. Third, although the search covered publications from 2010 to 2025, database indexing is dynamic, and late-indexed publications may affect annual production counts. Fourth, bibliometric maps are sensitive to keyword harmonization decisions. To reduce this bias, DOI and title normalization, keyword standardization, and a stop-word strategy were applied; however, residual variation in terminology may remain, particularly for threshold concepts such as LT1, LT2, VT1, VT2, RCP, and anaerobic threshold. Fifth, citation-based indicators are influenced by database coverage, publication age, and field-specific citation practices. Finally, this study maps the structure and evolution of the literature but does not evaluate the methodological quality or physiological validity of individual empirical studies.

## 6 Conclusion

This study revealed that the literature on cardiopulmonary performance and physiological thresholds in sports sciences showed steady growth in both volume and structure between 2010 and 2025. The regression model with annual production growth and a high explanatory showed that the field has been going through a systematic process of institutionalization rather than random fluctuations.

Journal concentration and Bradford distribution revealed that the literature exhibits a distinct core-periphery structure, with knowledge consolidated around a limited number of high-impact publishing platforms. Conceptual network and thematic map analyses showed that cardiopulmonary performance research is structured around physiological threshold parameters and exercise-based mechanisms. Centrality measurements confirmed that these core concepts are not only thematically but also centrally positioned in terms of structural network dynamics. The representation of niche themes, such as isocapnic buffering, as lower-level metabolic regulation mechanisms suggests that the field does not exhibit a peripheral weakness but rather has gone through a process of mechanistic deepening. On the other hand, citation half-life analyses revealed that the literature exhibits both a current and cumulative knowledge structure, supporting an evolutionary developmental pattern in the field. Consequently, research on cardiopulmonary performance and physiological threshold is a globally organized, structurally consolidated, and conceptually centralized scientific field. By integrating production trends, database coverage comparison, keyword harmonization, thematic mapping, collaboration analysis, and citation half-life indicators, this study provides a reproducible bibliometric framework for understanding the intellectual structure and future research directions of cardiopulmonary performance and threshold research in sports sciences.

## Data Availability

The processed dataset, variable descriptions, and analysis scripts supporting the findings of this study are publicly available in Zenodo at: https://zenodo.org/records/20665601.
