# Supplementary material for: The global scientific evolution of cardiopulmonary performance and threshold research in sports sciences during 2010–2025: a comparative bibliometric analysis based on web of science and scopus
Source: Front Res Metr Anal. 2026 Aug 3;11:1905302. doi: 10.3389/frma.2026.1905302 (PMC13478221; doi:10.3389/frma.2026.1905302)
Supplement: Supplementary file 1 [file Table_1.docx]

| **Database** | **Field** | **Exact search string** | **Filters** | **Search date** | **Records** |
| --- | --- | --- | --- | --- | --- |
| Scopus | TITLE-ABS-KEY | full syntax | Article; English; 2010–2025 | 25 Jun 2026 | Initial records: n = 2061; after filters: n = 1849 |
| Web of Science | TS | full syntax | Article; English; 2010–2025 | 25 Jun 2026 | Initial records: n = 1679; after filters: n = 1558 |

**Supplementary Table S1**. Complete search strategy used in Scopus and Web of Science Core Collection
